# A comparison of R-EPOCH and R-CHOP as a first-line regimen in *de novo* DLBCL patients with high Ki-67 expression in a single institution

**DOI:** 10.18632/oncotarget.9271

**Published:** 2016-05-10

**Authors:** Jia-Jia Huang, Yi Xia, Yu Wang, Pan-Pan Liu, Xi-Wen Bi, Peng Sun, Tong-Yu Lin, Wen-Qi Jiang, Zhi-Ming Li

**Affiliations:** ^1^ State Key Laboratory of Oncology in South China, Collaborative Innovation Center for Cancer Medicine, Sun Yat-Sen University Cancer Center, Guangzhou, China; ^2^ Department of Medical Oncology, Sun Yat-Sen University Cancer Center, Guangzhou, China

**Keywords:** R-EPOCH, DLBCL, Ki-67 expression, R-CHOP

## Abstract

Diffuse large B-cell lymphoma (DLBCL) patients with high Ki-67 expression receive limited benefits from R-CHOP (rituximab, cyclophosphamide, doxorubicin, vincristine, and prednisone) therapy. This study aims to compare the R-EPOCH (etoposide, prednisone, vincristine, cyclophosphamide, and doxorubicin) and R-CHOP regimens as first-line therapy in DLBCL patients with high Ki-67 expression. Data from 44 untreated DLBCL patients with high Ki-67 expression receiving R-EPOCH therapy were matched with those from 132 untreated DLBCL patients with high Ki-67 expression receiving R-CHOP therapy based on the International Prognostic Index (IPI: age, Ann Arbor stage, performance status, LDH level, number of extranodal sites), gender, and Ki-67 expression. In the R-EPOCH group, 42/44 patients were eligible for response evaluation. A total of 35 patients (83.3%) achieved complete remission (CR); 6 patients (14.3%) achieved partial remission (PR); and one patient (2.4%) exhibited progressive disease (PD) after 2 cycles of therapy. Patients in the R-EPOCH group presented better survival outcomes than those in the R-CHOP group (3-year overall survival [OS]: 89.9% vs. 70.2%, *p* = 0.041; 3-year progression-free survival [PFS]: 86.6% vs. 59.7%, *p* = 0.024). The survival superiority of the R-EPOCH over the R-CHOP regimen persisted when considering only patients of low-to-intermediate IPI risk, but it was not observed in those of high IPI risk. Our data suggest that R-EPOCH could be superior to R-CHOP as a first-line regimen in DLBCL patients with high Ki-67 expression, especially in those of low-to-intermediate IPI risk.

## INTRODUCTION

Diffuse large B-cell lymphoma (DLBCL) is the most common subtype of non-Hodgkin lymphoma (NHL), accounting for 30%–40% of all NHL patients [1–3]. DLBCL is considered to be a heterogeneous entity based on its biological characteristics and clinical outcomes [3–5]. R-CHOP (rituximab, cyclophosphamide, doxorubicin, vincristine, and prednisone) is the first-line standard treatment for DLBCL because the addition of rituximab to CHOP chemotherapy notably improves survival outcomes [6, 7]. However, some DLBCL patients continue to present an inferior prognosis under standard R-CHOP therapy. Therefore, many studies have been performed in an attempt to improve the current treatment for DLBCL with a poor prognosis [8–10].

For tumors with high proliferation, the EPOCH (etoposide, prednisone, vincristine, cyclophosphamide, and doxorubicin) regimen is based on the concept that the extension of drug exposure may yield better antitumor efficacy than a bolus regimen, such as CHOP [11–13]. Ki-67, a useful prognostic factor in various neoplasms, is considered to be a proliferation index [14, 15]. In a previous study, we found that DLBCL patients with high Ki-67 expression received limited survival benefits from R-CHOP therapy [16]. Hence, the present study aimed to investigate whether R-EPOCH is superior to R-CHOP in untreated DLBCL patients with high Ki-67 expression.

## RESULTS

### Patient characteristics

A total of 44 DLBCL patients with high Ki-67 expression in the study group treated with R-EPOCH and 132 DLBCL patients with high Ki-67 expression in the control group treated with R-CHOP were compared via matched-pair analysis. The clinical characteristics of all patients in both groups are shown in Table 1. The median age of the study group was 46 years (range: 19–69 years) and 48 years (range: 21–74) in the control group. An elevated LDH level and advanced disease (Ann Arbor stage III–IV) were found in 45% and 43% of the patients in the two groups, respectively. IPI score of 0–3 was observed in 82% of the patients in both groups. Bulky disease was present in 8 patients (18%) from the study cohort and 23 patients (17.4%) from the control cohort. The main clinical features of the patients were comparable in the study and control groups.

**Table 1:** Patient characteristics in the R-EPOCH and R-CHOP groups

Characteristics	Total (*N* = 176)	R-EPOCH group (*N* = 44)	R-CHOP group (*N* = 132)	*P*-value
Age (years)				
≤ 60	148	37	111	1.0
≥ 60	28	7	21	
Gender				
Male	80	20	60	1.0
Female	96	24	72	
Ann Arbor Stage				
I–II	76	19	57	1.0
III–IV	100	25	75	
B symptoms				
Absent	115	29	86	0.927
Present	61	15	46	
ECOG performance status				
0–1	144	36	108	1.0
≥ 2	32	8	24	
LDH level				
Normal	80	20	60	1.0
Elevated	96	24	72	
Bulky disease				
No	144	36	109	0.909
Yes	32	8	23	
IPI score				
0–3	144	36	108	1.0
4–5	32	8	24	

Abbreviations: ECOG PS, Eastern Cooperative Oncology Group performance status; LDH, lactate dehydrogenase;IPI, International Prognostic Index.

All patients and control subjects included in the study exhibited high Ki-67 expression (≥ 80%). The Ki-67 expression status (80%–90% vs. > 90%) was one of the matching variables in both the study group and the control group. A total of 75% of all patients in the R-EPOCH group (33 cases) and in the R-CHOP group (99 cases) exhibited Ki-67 expression, ranging from 80%–90%. Approximately three-fourths (26 cases, 76%) of the patients in the study group showed positive bcl-2 expression. The non-GCB subtype was found in 16 patients (18/34, 53%) and 45 patients (58/103, 56%) in the R-EPOCH and R-CHOP groups, respectively. The immunohistochemical expression of biomarkers in the R-EPOCH and R-CHOP groups is summarized in Table 2. No significant difference in the unmatched clinical features and biomarker expression was observed in the R-EPOCH and R-CHOP groups.

**Table 2:** Biomarkers in the R-EPOCH and R-CHOP groups

Biomarkers	Total (No. evaluated)	R-EPOCH group (No. evaluated)	R-CHOP group (No. evaluated)	*P*-value
Ki-67	176	44	132	1.0
≤ 90%	132	33	99	
> 90%	44	11	33	
BCL-2	149	34	115	0.690
Negative	39	8	31	
Positive	110	26	84	
DLBCL subtype	137	34	103	0.732
GCB	61	16	45	
Non-GCB	76	18	58	
BCL-6	139	36	103	0.627
Negative	51	12	39	
Positive	88	24	64	
CD10	151	36	115	
Negative	93	21	72	0.645
Positive	58	15	43	
Mum-1	131	32	99	
Negative	40	10	30	0.919
Positive	91	22	69	

### Treatment outcomes and toxicity in the R-EPOCH group

In the R-EPOCH group, 42 patients (95.5%) were eligible for response evaluation. Complete remission (CR) was achieved in 35 patients (83.3%), and partial remission was achieved in 6 patients (14.3%). One patient exhibited disease progression after 2 cycles of R-EPOCH therapy. Within a median follow-up of 30.6 months (range, 7.3–71.4 months), 3 patients died of progressive lymphoma, and one patient died of cardiovascular disease.

A total of 218 cycles of R-EPOCH therapy were administered, with a median of 4 cycles (range: 2 to 8 cycles). The major side effect of the R-EPOCH regimen was hematologic toxicity. Grade 3/4 neutropenia, anemia and thrombocytopenia were observed in 32.2% (70 cycles), 4.6% (10 cycles), and 9.2% (20 cycles) of the cycles, respectively. Neutropenic fever developed in 13.8% of the cycles (30 cycles). Mild peripheral neuropathy was present in approximately one-third of the patients (13 patients, 29.5%) but was mild and controllable. The observed gastrointestinal toxicity, which included vomiting, mucositis and constipation, was mild to moderate and manageable. Cardiac toxicity from epirubicin (EPI) or pirarubicin (THP) did not exhibit any significant impact on R-EPOCH administration. No patients exhibited a decrease in the cardiac ejection fraction leading to a discontinuation of EPI (or THP) or the development of congestive heart failure. No treatment-related deaths were observed in the R-EPOCH group.

### Survival outcomes and prognostic factors

**Figure 1:**
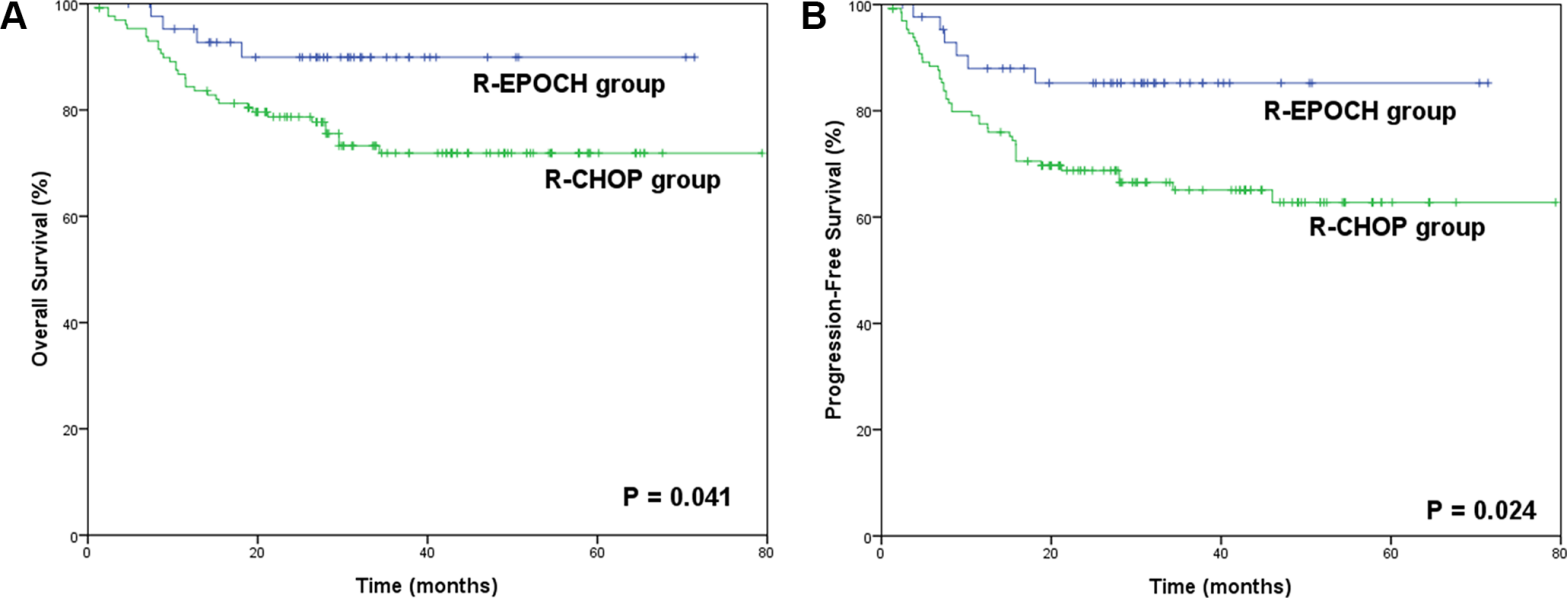
Survival outcomes in the R-EPOCH and R-CHOP groups (**A**) Overall survival (OS) in the R-EPOCH and R-CHOP groups. (**B**) Progression-free survival (PFS) in the R-EPOCH and R-CHOP groups.

**Figure 2:**
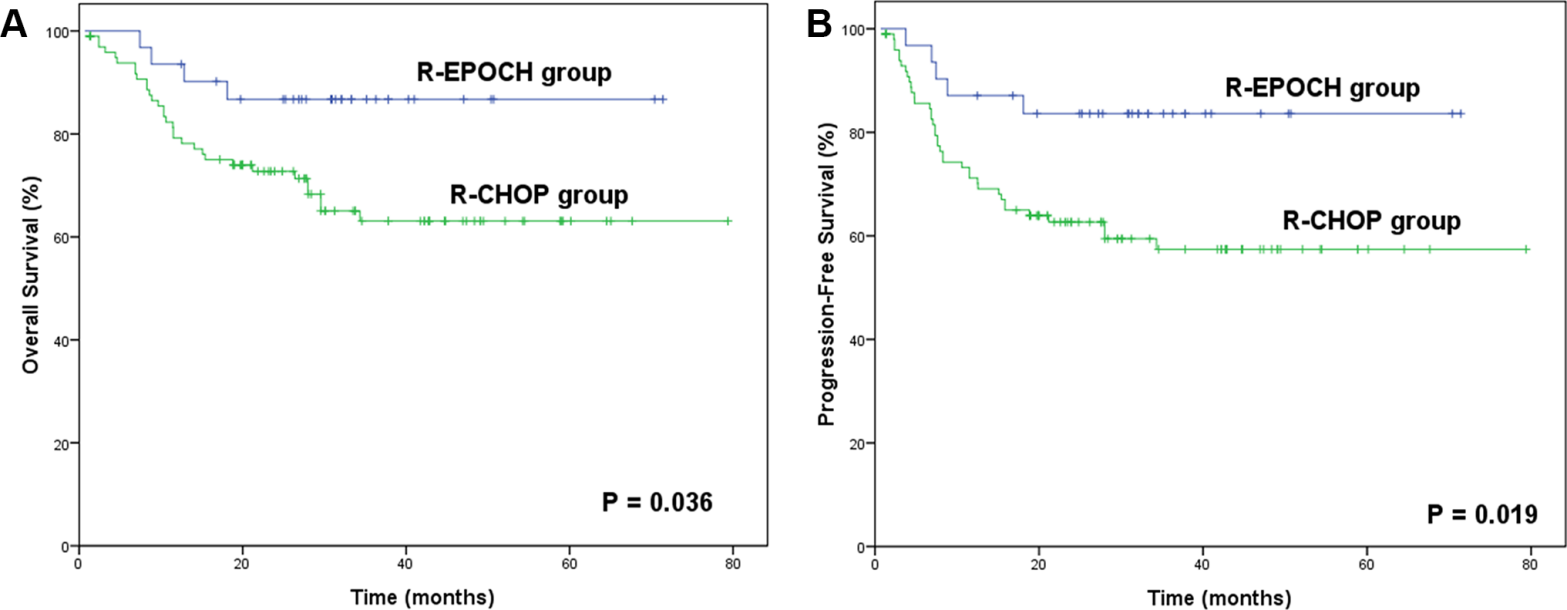
Survival outcomes in the R-EPOCH and R-CHOP groups according to the Ki-67 expression status (**A**) Overall survival (OS) in the R-EPOCH and R-CHOP groups with Ki-67 expression of 80%–90%. (**B**) Progression-free survival (PFS) in the R-EPOCH and R-CHOP groups with Ki-67 expression greater than 90%.

**Figure 3:**
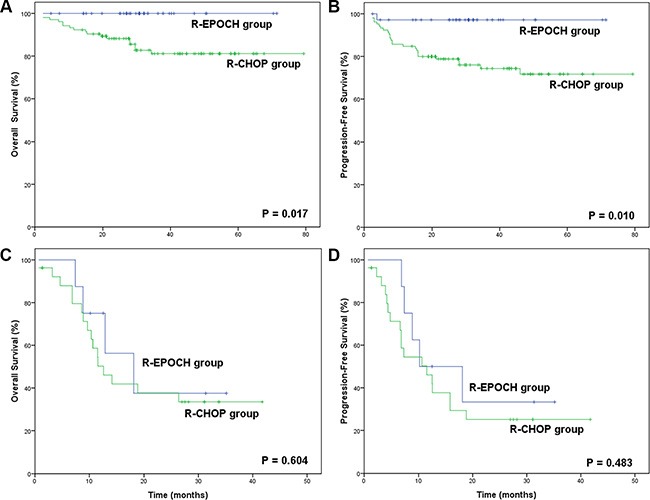
Survival outcomes in the R-EPOCH and R-CHOP groups according to the International Prognostic Index (IPI) (**A**) Overall survival (OS) in the R-EPOCH and R-CHOP groups with low-to-intermediate IPI risk. (**B**) Progression-free survival (PFS) in the R-EPOCH and R-CHOP groups with low-to-intermediate IPI risk. (**C**) Overall survival (OS) in the R-EPOCH and R-CHOP groups with high IPI risk. (**D**) Progression-free survival (PFS) in the R-EPOCH and R-CHOP groups with high IPI risk.

In the R-CHOP group, the 3-year OS and PFS rates were 70.2% and 59.7%, respectively. The patients in the R-EPOCH group presented superior survival outcomes over those in the R-CHOP group (3-year OS: 89.9% vs. 70.2%, *p* = 0.041; 3-year PFS: 86.6% vs. 59.7%, *p* = 0.024), as shown in Figure 1. The survival superiority of the R-EPOCH regimen over the R-CHOP regimen remained in patients who showed Ki-67 expression of 80%–90% (3-year OS: 86.7% vs. 63.1%, *p* = 0.036; 3-year PFS: 83.6% vs. 57.4%, *p* = 0.019, as indicated in Figure 2), but not in patients who showed Ki-67 expression > 90% (*p* = 0.719 in OS, and *p* = 0.745 in PFS). Figure 3 shows the comparison of survival outcomes in the R-EPOCH and R-CHOP groups according to IPI risk. In patients with a low-to-intermediate-risk IPI (IPI score of 0–3), the R-EPOCH regimen resulted in better survival outcomes than did the R-CHOP regimen (3-year OS: 100% vs. 81.1%, *p* = 0.017; 3-year PFS: 97.1% vs. 74.3%, *p* = 0.010). However, no survival benefit was found in patients with a high-risk IPI (IPI score: 4–5) treated with the R-EPOCH regimen compared with those with a high-risk IPI treated with the R-CHOP regimen (3-year OS: 37.5% vs. 35.5%, *p* = 0.604; 3-year PFS: 33.3% vs. 25.1%, *p* = 0.483).

Table 3 lists the results of the univariate analysis of prognostic factors for survival outcomes in the R-EPOCH group. The following variables were found to have an adverse impact on survival outcomes: high-risk IPI (*p* < 0.001 in both OS and PFS), bulky disease (*p* < 0.001 in both OS and PFS) and B symptoms (*p* = 0.002 in OS, and *p* = 0.019 in PFS). Due to the limited sample size of the R-EPOCH group, multivariate analysis was not performed further.

**Table 3:** Univariate analysis of prognostic factors for survival in the R-EPOCH group

Parameters	Overall survival (OS) *P*-value	Progression-free survival (PFS) *P*-value
IPI score (0–3 vs. 4–5)	< 0.001	< 0.001
Bulky disease	< 0.001	< 0.001
B symptoms	0.002	0.019
Bcl-2 expression status	0.256	0.203
CD10 expression status	0.534	0.317
Bcl-6 expression status	0.526	0.209
Mum-1 expression status	0.915	0.590
DLBCL subtype	0.385	0.202

Abbreviations: IPI, International Prognostic Index; DLBCL, diffuse large B-cell lymphoma.

## DISCUSSION

Rituximab, which targets the CD20 antigen, was the first monoclonal antibody approved for use in patients with lymphoma [7]. The combination of rituximab and CHOP chemotherapy showed additional benefits in DLBCL patients in randomized controlled trials [17–20]. R-CHOP has been adopted as the standard first-line therapy for DLBCL [6]. However, DLBCL is an entity that presents heterogeneous biological characteristics and clinical behaviors [5]. Patients who exhibit poor clinical outcomes under standard R-CHOP therapy pose a difficult challenge. Many studies have made attempts to explore novel biological markers for identifying the patients who would receive limited benefits from R-CHOP therapy [16, 21–23].

Ki-67, a surrogate marker of proliferation, has been investigated in various neoplasms and found to be a powerful prognostic factor for survival outcomes [14, 16, 24–26]. Patients with highly proliferative tumors show much poorer survival than those with tumors characterized by low proliferation [24]. In a previous study, we investigated Ki-67 expression in DLBCL patients in the era of rituximab treatment. Our results indicated that high Ki-67 expression was associated with adverse clinical behaviors. Patients with a non-GCB subtype with high Ki-67 expression receive limited survival benefits from R-CHOP therapy [16]. Therefore, determining the optimal treatment for DLBCL patients with high Ki-67 expression remains a challenge.

The EPOCH regimen was designed based on experimental findings showing that continuous low-concentration exposure to drugs could enhance the effectiveness of cell-killing in malignant cells with high proliferation [11, 27, 28]. In addition, *in vitro* studies suggested that prolonged low-dose drug exposure could overcome the resistance mediated by MDR-1 in tumor cells [29]. The EPOCH regimen has shown promising results and safe profiles in relapse or refractory non-Hodgkin lymphomas [29–32]. The combination of the EPOCH regimen (or the dose-adjusted regimen) and rituximab has also been evaluated in several clinical trials [23–25]. Here, we administered R-EPOCH as a first-line regimen in DLBCL patients with high Ki-67 expression and compared the treatment efficacy of R-EPOCH and R-CHOP therapy in this subgroup using matched-pair controls. Our results suggested that patients treated with the R-EPOCH regimen exhibited better survival than those administered the R-CHOP regimen. The superiority of the R-EPOCH regimen persisted in patients showing Ki-67 expression of 80%–90% but not in patients exhibiting Ki-67 expression > 90%. The main reason for this result lies in the small sample size of patients showing Ki-67 expression > 90% (25%, 11 cases). Whether the R-EPOCH regimen shows better efficacy than the R-CHOP regimen in DLBCL patients with Ki-67 expression > 90% needs to be evaluated in a much larger population. When patients were stratified by IPI risk, it was found that the patients with a low-to-intermediate IPI risk received better survival benefits from the R-EPOCH regimen than the R-CHOP regimen. There were only 8 cases in the high-risk IPI group, and no significant difference in survival outcomes was found in the R-EPOCH and R-CHOP groups. The limited number of patients in the high-risk group might be one of the reasons for these negative results. The efficacy of R-EPOCH regimen in high-risk DLBCL patients is still uncertain [10, 36], which needs to be explored in the prospective studies. A phase III randomized study of comparison R-CHOP and R-EPOCH regimen in treating DLBCL in the US is still ongoing, and we are expecting the final results.

Certain biomarkers have been assessed to determine their relationship with survival outcomes in DLBCL patients treated with a dose-adjusted R-EPOCH regimen, such as bcl-2, bcl-6, and the GCB subtype [33, 35, 37]. However, the results have been controversial [33, 35, 37]. In the present study, common pathological biomarkers were also evaluated through univariate analysis, but no association with survival was found.

In conclusion, R-EPOCH could be superior to R-CHOP as a first-line regimen in DLBCL patients with high Ki-67 expression, particularly in those of low-to-intermediate IPI risk. Further prospective studies are warranted to confirm our findings and to identify possible prognostic biomarkers for use in association with R-EPOCH therapy.

## MATERIALS AND METHODS

### Patients and study design

Our cohort included 44 patients with untreated de novo DLBCL diagnosed at Sun Yat-Sen University Cancer Center, China, from May 2005 to October 2012. The patients included in this study fulfilled the following criteria: (1) histologically proven diagnosis of DLBCL with positive expression of CD20, according to the WHO classification of Tumours of Haematopoietic and Lymphoid Tissues [38]; (2) Ki-67 immunohistochemical expression ≥ 80%; (3) no previous treatment; (4) no previous neoplasm or second malignancy; (5) no severe coincident disease; and (6) available clinical information and follow-up data. Patients with primary central nervous system lymphoma and those with human immunodeficiency virus infection or DLBCL secondary to low-grade lymphoma were excluded from this cohort. Grey zone lymphoma and composite lymphoma were also excluded from this study. Antibodies to the following antigens were evaluated for immunophenotype analysis: CD10, Bcl-6, MUM1/IRF4, Bcl-2, CD20, CD79α, and CD3. Germinal center B-cell (GCB) and non-GCB DLBCL subtypes were classified based on the algorithm proposed by Hans et al. [39]. This study was performed in accordance with the Declaration of Helsinki, and it was approved by the Institutional Review Board (IRB) of Sun Yat-Sen University Cancer Center. Written informed consent was obtained from all patients before the collection of patients' information. The clinical available data included patient demographics, physical examination results, Eastern Cooperative Oncology Group (ECOG) performance status (PS), B symptoms, serum lactate dehydrogenase (LDH) levels, bone marrow examination results, and computed tomography (CT) or positron emission tomography/CT (PET/CT) scans. All patients were staged according to the Ann Arbor Staging system and analyzed using the International Prognostic Index (IPI: age, PS, stage, LDH level, and extranodal sites).

In the matched-pair analysis performed in this study, patients treated with R-EPOCH were matched to those receiving R-CHOP therapy during the same period at a ratio of 1:3. The source of the matching control group was 836 consecutive de novo DLBCL patients treated with R-CHOP as a first-line therapy at Sun Yat-Sen University between May 2005 and October 2012. The patients were matched for the following variables: gender (male vs. female), age (± 5 years), Ann Arbor stage (I–II vs. III–IV), ECOG PS (0–1 vs. 2–3), LDH levels (normal vs. elevated), extranodal sites (0–1 vs. ≥ 2), and Ki-67 expression (80%–90% vs. > 90%). All of the study cohort and the control group in the matched-pair analysis exhibited high Ki-67 expression (≥ 80%). All of the above factors were fully matched among the study cases and the three controls. If a case could be matched with more than 3 controls, the 3 controls were selected randomly.

### Treatment and response criteria

All 44 patients in the study cohort received an EPOCH regimen combined with rituximab as first-line chemotherapy for 2 to 8 cycles (median, 6 cycles). Rituximab was administered on day 1 at a dose of 375 mg/m^2^. The EPOCH regimen included doxorubicin (10 mg/m^2^, continuous intravenous infusion, days 2 to 5), etoposide (50 mg/m^2^, continuous intravenous infusion, days 2 to 5), vincristine (0.4 mg/m^2^, continuous intravenous infusion, days 2 to 5), cyclophosphamide (750 mg/m^2^, intravenous bolus, day 6), and prednisone (60 mg/m^2^, orally, days 2 to 6). The R-EPOCH regimen was administered every 21 days.

In the matching control group, all patients were treated with R-CHOP as first-line therapy for 2 to 8 cycles (median 6 cycles). The administration of rituximab was as described above in the R-EPOCH regimen. The CHOP regimen included cyclophosphamide (750 mg/m^2^, day 2), doxorubicin (50 mg/m^2^, day 2), vincristine (1.4 mg/m^2^, at a maximal dose of 2 mg, day 2) and prednisone (60 mg/m^2^, days 2 to 6). The treatment schedule was repeated every 21 days.

In both the study cohort and the matching control group, involved field radiation (30–56 Gy) was delivered to the residual disease, extranodal sites, or previous bulky disease via a conventional fractionation scheme (daily fraction of 2 Gy, 5 fraction per week) after the chemotherapy.

The response to treatment was assessed according to the International Working Group Recommendation for Response Criteria for non-Hodgkin lymphoma [40, 41]. The evaluation of adverse effects after chemotherapy was based on the National Cancer Institute (NCI) criteria [42].

### Immunohistochemistry for Ki-67

Immunohistochemical analysis of Ki-67 was carried out using a mouse monoclonal anti-Ki-67 antibody (1:100; Invitrogen, Carlsbad, CA, USA). Formalin-fixed, paraffin-embedded sections (4 μm thick) were deparaffinized and rehydrated through a graded series of alcohols. The immunohistochemical method for Ki-67 detection was performed as previously reported [16]. Ki-67 expression was detected in the nucleus of tumor cells. The proportion of cells showing Ki-67 expression was evaluated based on the number of lymphoma cells with nuclear immunoreactivity and the total number of tumor cells in the highest labeling field at high magnification (400 ×). High Ki-67 expression was considered to be present in this study when antibody staining for Ki-67 in the nucleus was observed in 80% or more of the lymphoma cells. Evaluation of the immunostaining and cell counts was performed independently at diagnosis by two pathologists who were blinded to the clinical outcomes of the patients.

### Statistical analysis

The primary aim of this study was to compare the treatment efficacy of R-EPOCH and R-CHOP therapy as first-line regimens in DLBCL patients with high Ki-67 expression. Overall survival (OS) and progression-free survival (PFS) were the primary end points of this study. OS was calculated from the date of diagnosis to the date of death due to any cause or to the date of the last follow-up. PFS was calculated from the date of first progression, relapse, death, or the last follow-up. The Pearson's Chi-squared test was used to compare the categorical variables. Survival curves and univariate analysis were performed via the Kaplan-Meier method. Differences were determined using a two-tailed log-rank test, and *p* < 0.05 was considered statistically significant. Statistical analysis was carried out with SPSS 16.0 software.
